# Supplementary material for: Are carbon emissions trading and green financial instruments synergistic? -Comprehensive quantitative research based on content analysis
Source: PLoS One. 2024 Mar 7;19(3):e0298601. doi: 10.1371/journal.pone.0298601 (PMC10919601; doi:10.1371/journal.pone.0298601)
Supplement: S1 Data — (ZIP) [file pone.0298601.s001.zip › Data/地级市Z_F工作报告.zip!/║╙─╧/╨┼╤⌠╩╨/└┤╫╘╣1⁄2╓┌║┼í╛┬φ┐╦╩2╛▌═°í┐_2015.docx]

各位代表：

　　现在，我代表信阳市人民政府，向大会报告工作，请予审议，并请各位政协委员和其他列席人士提出意见。

　　一、2014年工作回顾

　　过去的一年，面对严峻复杂的形势和艰巨繁重的任务，在省委、省政府和市委的正确领导下，全市认真贯彻落实党的十八大和十八届三中、四中全会精神，深入开展党的群众路线教育实践活动，坚持持续求进、务实发展、积极作为，统筹稳增长、促改革、调结构、强支撑、控风险、惠民生，较好地完成了市四届人大四次会议确定的各项任务。

　　初步核算，全市生产总值1757.34亿元，增长8.9%；规模以上工业增加值499.81亿元，增长12.3%；固定资产投资1723.17亿元，增长19.1%；社会消费品零售总额761.43亿元，增长12.8%；财政总收入107.66亿元，增长15.6%，一般公共预算收入80.33亿元，增长18.2%；城镇居民人均可支配收入21060元、农民人均纯收入8868元，分别实际增长8.1%和9.2%；居民消费价格上涨1.7%；节能减排目标全面完成。我市在全面建成小康社会的进程中迈出了新的步伐。

　　一年来，我们主要做了以下工作：

　　（一）着力促增长调结构，发展质量有新提升。坚持以扩投资为促增长提供支撑，以促增长为调结构创造条件，促进经济持续健康发展。积极扩大需求。坚持把发展现代工业作为补短板、调结构的主攻点来抓，组织实施工业稳增长调结构促发展活动，全年完成工业投资629.19亿元，增长33.5%，增速居全省第1位。实施194个重大工业结构调整项目，已有65个项目建成投产；坚持把发展现代服务业作为扩消费的支撑点来抓，出台了加快发展服务业、电子商务、信息消费等实施意见，大力发展金融服务、信息服务、文化旅游等高成长服务业和健康养老、科技服务、电子商务、物流快递等新兴服务业，持续推进房地产业健康发展，启动创建国家5A级旅游景区和国家生态旅游示范区工作。全市第三产业增加值587.5亿元，增长10.3%，总量占生产总值的比重达到33.4%，较上年提高0.4个百分点；坚持把扩大出口作为促增长的突破点来抓，认真落实国家支持外贸转型升级促进对外贸易健康发展的政策措施，全市出口总值2.9亿美元，增长36.8%，增速居全省第4位；坚持把做优农业作为促增长的基础来抓，深入实施 “三农”四大工程，发放各类惠农补贴16.1亿元，新建高标准粮田79.2万亩，全年粮食总产量118.44亿斤，新增1.23亿斤，实现“十一连增”。全市畜牧业健康发展，肉蛋奶产量稳定增长。积极发展新型农业经营主体，农民专业合作社达到7277家，家庭农场1454家，已建成农业产业化集群28个，市级以上农业产业化重点龙头企业375家。强化要素保障。全面优化金融生态环境，促进金融机构支持实体经济发展，积极推动直接融资。去年年底，全市金融机构各项存款余额2094.96亿元，各项贷款余额1107.75亿元，全年新增贷款198.3亿元，实现资本市场融资66.4亿元。稳步推进人地挂钩政策试点，充分运用城乡建设用地增减挂钩政策，争取省政府批准建设用地2.1万亩，有效保障了发展需求。提前完成信阳电网“十二五”规划“1230”工程，全市供电能力大幅提升。深入实施全民技能振兴和职教攻坚二期工程，各类职业技能培训17.9万人次，较好满足了企业用工需求。务实服务企业。着力加强产销对接、用工对接、银企对接，力促市场前景好的企业扩大生产，帮助困难行业和企业降本增效、开拓市场，扶持中小微企业加快发展。全市共有5家企业进入全省百高、百强企业行列，河南黄国粮业在“新三板”成功上市。全市规模以上工业利税、利润总额分别增长14.6%和21.7%。

　　（二）着力强载体建体系，发展能力有新提升。突出抓好“一个载体、四个体系”，着力增强经济发展的科学性和可持续性。加快产业集聚区建设。大力实施产业集聚区“四大工程”、“三大计划”，认真落实重点项目攻坚督导协调例会制度，加快108个重点攻坚项目建设。全市产业集聚区完成固定资产投资963.8亿元，增长26.2%；产业集聚区规模以上工业主营业务收入突破1500亿元，占全市工业主营业务收入的75%，产业集聚区的综合带动作用日益增强。加快构建现代产业体系。集中要素资源，扶持壮大高成长性产业、改造提升传统优势产业、培育发展战略新兴产业。我市电子信息、新型建材、绿色食品、纺织服装等主导产业初具规模，现代家居、现代物流等产业集群集聚态势正在形成。其中电子信息、纺织服装产业增加值分别增长72.7%和18.3%，增速分别高于全市规模以上工业增加值60.4和6个百分点。加快构建现代城乡体系。组织修编了《信阳市城市总体规划（2014－2030年）》。大力实施新型城镇化三年行动计划，统筹推进中心城市、县城和中心镇协调发展，加快推进城乡基础设施一体化建设，全市交通、电力、通信等基础设施进一步完善，中心城区地下管网和一批主次干道得到改造提升，内河治理、垃圾处理、污水处理等工程持续实施，教育、卫生、文化、体育等公共服务设施布局进一步优化，公共自行车系统在部分区域开通运营。强化“一基本两牵动三保障”，有序促进农村劳动力进城转移，全市城镇化率达到41.1%。深入推进数字城市、智慧城市建设，城市精细化、数字化、市场化管理不断加强。加快构建自主创新体系。顺利实施一批重大科技专项，成功组建国家稻米精深加工产业技术创新战略联盟和河南省茶产业技术创新战略联盟。全市省级工程技术研究中心达到25家，院士工作站6家，高新技术企业14家。全市高新技术产业增加值增长19.4%，高于规模以上工业增加值增速7.1个百分点。大力实施商标战略、名牌战略和质量立市战略，全市已获得中国驰名商标9件、中国地理标志证明商标5件，已有3家企业获得省长质量奖、12家企业获得市长质量奖。加快构建现代市场体系。落实工商登记便利化，全市培育各类市场主体16.1万户，增长25.4%。积极构建新兴市场体系，全市商务中心区和特色商业区累计完成投资130亿元，市商务中心区入驻企业153家；大力推进商品交易市场建设，规划建设了国际茶城、国际商城等一批专业市场，信阳被农业部批准为全国唯一的国家级茶叶市场；深入推进“万村千乡市场工程”建设，新建了一批社区便民蔬菜超市；着力完善要素市场，构建了人力资源“网上超市”、劳动力“招聘集市”、基层就业和社保“服务门市”，建设了公共资源交易中心、国土资源交易中心和金融服务中心。

　　（三）着力打基础增优势，发展后劲有新提升。围绕重点领域和关键环节，着力打基础增后劲，发展优势不断提升。加强基础设施建设。境内的312和107国道升级改造工程、宁西铁路复线电气化改造和京港澳高速公路改扩建工程进展顺利；淮河航运基础设施建设加快推进；信阳火车站改扩建和信阳东站东甬道工程获得中铁总公司批准；明港机场即将开工建设。统筹推进“三件大事”。集中力量抓好三大项目建设，电子信息产业集聚示范区已集聚电子信息企业86家，初步形成智能通信终端、智能显示终端两大产业链和智能手机、伯皇IT两大产业园，被中国通讯工业协会命名为“中国手机产业基地”；国际家居产业小镇累计完成投资50亿元，基本完成一期基础设施配套，已开工建设企业22家，建成投产或运营3家；信阳义乌国际商贸城正在有序推进。积极谋划重大专题。信阳被确定为首批国家主体功能区建设试点示范市和全国第二批农村改革试验区，大别山革命老区振兴发展规划和淮河生态经济带发展规划上升到国家战略研究层面。

　　（四）着力抓改革促开放，发展活力有新提升。坚持用改革的办法和开放的途径破解发展难题，发展动力活力不断增强。全面深化各项改革。认真落实中央和省委全面深化改革的统一部署，出台了《信阳市全面深化改革实施意见》，第一批重点改革事项稳步推进。农村改革取得新的突破，深化集体林权制度改革、改进农业补贴办法、涉农建设性资金整合、扶贫开发综合改革等被确定为全国试点；持续推进行政审批制度改革，市本级取消行政审批项目53项，合并与下放行政审批项目13项；持续深化国有企业改革，有序推进国有粮食企业产权制度改革，市直机关所属企业脱钩改制工作取得积极进展；稳步推进政府机构改革，工商、质监、药监等体制调整顺利实施，初步完成事业单位分类改革前期工作；财税、投融资和金融改革有序推进，户籍、林权、水利、供销、农电和社会事业体制改革等全面展开，一些重要领域和关键环节改革取得重大进展。大力推进开放招商。持续深化“四一”招商行动，全市实际利用外商直接投资4.8亿美元，增长13.2%。成功举办信阳第22届国际茶文化节、第九届中国仓储业大会，信阳对外开放水平不断提升。成功举办“中国光彩事业信阳行”活动，已落地项目126个，完成投资425亿元。全面推进战略合作，信阳与北京市西城区缔结为友好市区，与部分省直单位签订了一批战略合作协议，五粮液集团对河南乌龙酒业成功实施战略重组。

　　（五）着力建设生态文明，发展水平有新提升。坚持把提升信阳生态优势作为可持续发展的最大竞争力来抓，把生态文明理念融入城市建设和经济社会发展全过程。加强生态环境保护。淮河源生物多样性保护和可持续利用项目圆满完成，顺利通过联合国开发计划署终期评估。大力实施林业生态市建设提升工程，深入创建全国绿化模范城市，加快构建绿色生态体系，不断提升生态自然修复能力。全年完成造林34.9万亩、森林抚育和改造37.2万亩。全市森林覆盖率达到35.2%。加强环境综合治理。大力实施“蓝天工程”、“碧水工程”、“乡村清洁工程”，突出抓好重点行业企业的大气污染治理、重点区域大气环境深度治理、城市低空面源污染治理和机动车污染治理，继续推进南湾湖国家重点湖泊生态保护和环境治理，加强农村面源污染和土壤污染治理，超额完成黄标车和老旧车淘汰任务，信阳环境质量持续位居全省前列。加强节能减排工作。出台了信阳市产业发展和区域开发环境保护负面清单，实行更加严格的环境准入制度和固定资产投资节能评估审查制度，全面推进节能降耗行动计划，大力实施一批循环经济、清洁生产和节能降耗示范工程，推进资源节约集约利用，用硬措施完成了节能减排的硬任务。加快美丽信阳建设。坚持山水林城一体发展，严禁破坏山体、园林、绿带和水系，进一步提升了“山水信阳·休闲茶都”的城市品牌。大力改善城乡环境，新建改建农村公路1440公里；新增燃气用户32059户，新解决54.2万农村居民和17.2万农村学校师生饮水安全问题，让更多家庭用上了清洁能源、喝上了干净卫生的自来水。扎实推进美丽乡村建设，全市有15个镇被命名为国家重点镇，4个乡镇被命名为国家级生态乡镇，4个村被确定为中国美丽乡村首批创建试点，12个村被列入中国传统村落名录。

　　（六）着力保底线惠民生，社会建设有新提升。坚持政府过紧日子思想，持续增加民生投入，全市用于民生支出252亿元，占公共财政支出的75.1%，有力保障了省 “十项重点民生工程”和市“十件实事”的扎实推进。努力促进就业创业。实施积极的就业扶持政策，更加有针对性地做好大学毕业生、农村转移劳动力、城镇困难群众、退役军人等群体就业帮扶工作，支持创业带动就业。全年城镇新增就业9.97万人，失业人员实现再就业3.58万人，就业困难人员实现就业1.49万人，让更多的人在“家门口”实现了就业梦。不断强化社会保障。城乡居民基本养老保险制度实现全覆盖，城镇职工和城乡居民基本养老保险关系实现了有效衔接，全年发放社会保险金43.6亿元；企业退休人员基本养老金连续十年递增10%，人均养老金水平达到1666元。失业、工伤、生育等保险覆盖面继续扩大。住房公积金事业稳步发展。社会救助制度全面建立，城乡低保对象人均年补助标准分别提高到3600元以上和1800元，农村五保集中供养和分散供养标准分别提高到3200元和2220 元，中心城区和各县最低工资标准分别提高到1250元和1100元，全年发放城乡低保和农村五保救助资金7.3亿元。实施精准扶贫，又有11万农村贫困人口实现稳定脱贫。统筹资金15.16亿元，基本建成保障性住房17107套、安置房1882套，改造农村危房25230户，又有一批城乡低收入家庭喜迁新居。大力发展社会事业。优先发展教育，全年财政用于教育支出80亿元。启动实施了学前教育第二个三年行动计划，“入园难”、“入园贵”问题得到有效缓解。继续改善农村贫困地区义务教育薄弱学校办学条件，义务教育持续均衡发展。高中教育、职业教育位居全省前列；大力发展卫生计生事业，公立医院综合改革全面启动，人均基本公共卫生经费提高到35元，新农合和城镇居民医保财政补助标准每人每年提高到320元。重大传染疾病防治不断加强。人口自然增长率控制在5.33‰以内；慈善、社会福利、老龄、妇女儿童、残疾人等事业全面发展；大力实施文化体育惠民工程，积极开展全民健身运动，成功举办全国自行车公开赛并被评为2014年中国体育旅游精品项目。积极创新社会治理。持续深化平安信阳、法治信阳建设，加强社会治安综合治理，依法打击各类刑事犯罪活动；实施依法逐级走访，创建民声手机短信平台，积极化解社会矛盾纠纷和信访积案；深入开展食品药品安全专项治理和安全生产专项整治，依法打击和处置非法集资，依法解决拖欠农民工工资问题，不断完善公共安全应急管理体系，群众安全感、满意度持续上升。

　　（七）着力抓整改转作风，发展环境有新提升。严格执行中央八项规定和省市若干意见，持之以恒纠正“四风”，全面营造风清气正的发展环境。大力推进依法治市，自觉接受人大监督、政协民主监督和社会监督，认真办理人大代表建议和政协提案，支持民主党派、工商联和无党派人士参政议政。加强依法行政和服务型行政执法，做好行政复议工作，推进社会信用体系建设。严格落实国务院“约法三章”，政府性楼堂馆所、机关事业单位人员编制得到有效控制，“三公”经费下降10%。强化廉政勤政，加强行政监察和审计监督，推进党风廉政建设和反腐败工作，严肃查处违纪违法案件，政府自身建设得到加强。

　　高度重视国防教育，不断加强国防后备力量及人民防空建设，深入开展双拥工作，推动军民融合深度发展，驻信解放军、武警部队和民兵预备役人员为地方发展作出了重要贡献。工会、共青团、妇联和科协、文联、社科联、红十字会等人民团体在经济社会发展中发挥了不可替代的作用，民族、宗教、外事、侨务、对台工作全面加强，统计、物价、机关事务、地质、气象、地震、人防、农机、档案、史志等工作取得了新成绩。

　　各位代表！一年来，我们坚持持续求进、务实发展、积极作为，全市生产总值、固定资产投资同时突破1700亿元，规模以上工业主营业务收入突破2000亿元，全市财政总收入突破100亿元，一般公共预算支出突破300亿元，金融机构各项存款余额突破2000亿元，各项贷款余额突破1100亿元。信阳加快发展的基础更加坚实。

　　一年来，我们坚持打基础利长远、兴产业带全局、重实效惠民生，出山店水库导流明渠工程开工建设，信阳海关通关运营，信阳东500千伏输变电工程建成投运，一批金融机构入驻信阳，一批主导产业初具规模，信息、水利、能源、生态环境等基础设施不断完善，多元化、优质化的金融服务市场逐步形成，发展优势、发展载体、体制机制等支撑条件有效提升，“一市一区两枢纽一基地”建设取得重大进展。信阳加快发展的支撑更加有力。

　　一年来，我们坚持在保护美的前提下建设美创造美，坚决不上高污染、高能耗、高排放项目，坚决不干违背规律、脱离实际的工程，坚决不做破坏生态环境、损害群众利益的事情。信阳成功创建国家卫生城市，成功再创省级文明城市并成为创建全国文明城市提名城市，再获中国最美丽城市，连续六届蝉联中国十佳宜居城市，连续四届蝉联中国最具幸福感城市。信阳加快发展的前景更加美好。

　　在外部环境偏紧、经济下行压力加大、困难挑战较多的情况下，取得这些成绩来之不易。这是省委、省政府和市委正确领导的结果，是人大、政协监督支持的结果，是全市上下团结拼搏的结果。在此，我代表市人民政府，向全市广大工人、农民、知识分子、干部职工，向驻信部队和武警官兵、公安干警，向中央和省属驻信单位以及社会各界人士致以崇高的敬意和衷心的感谢！

　　各位代表！在肯定成绩的同时，也要清醒地看到，我市经济社会发展中还存在一些突出矛盾和问题。一是经济快速增长的难度逐步加大。我市面临的宏观环境错综复杂，经济运行中的不确定、不稳定因素依然较多，部分行业和企业生产经营仍较困难，生产要素供应日益趋紧，经济下行压力依然较大。二是结构性矛盾依然突出。三产之间、三大需求之间结构不够协调，城乡之间、县区之间发展还不平衡，一些重要领域的体制机制障碍尚需破除，瓶颈制约因素依然较多。三是改善民生的任务依然艰巨。群众关注的事情还有一些没有完全办好，社会治安、安全生产、食品药品安全等领域还存在薄弱环节，群众对教育、医疗、养老、住房、征地拆迁安置等还有不少不满意的地方。四是发展环境有待进一步优化。政府职能转变、简政放权还不到位，到个别部门办事难、办事慢的问题还没从根本上解决，一些干部还存在“换挡焦虑”和“本领恐慌”，公务员队伍的综合素质有待于进一步提高。对此，我们一定自我加压，敢于担当，认真解决，决不辜负人民期望！

　　二、2015年政府工作的主要预期目标和重点工作

　　今年是全面深化改革的关键之年，是全面推进依法治国的开局之年，也是全面完成“十二五”规划的收官之年。做好今年的政府工作，意义重大。

　　按照市委经济工作会议的总体要求，今年经济社会发展的主要预期目标是：在优化结构、提高质量、改善环境基础上，生产总值增长8.5%左右，在实际工作中努力争取更好结果；一般公共预算收入增长12%；全社会固定资产投资增长16%；社会消费品零售总额增长12%；进出口总值增长9%；实际利用外商直接投资增长10%；城镇新增就业9万人以上；城乡居民收入增长与经济增长同步；居民消费价格涨幅3%以下；人口自然增长率控制在6.5‰以内；节能减排和环境质量改善完成省下达任务。

　　经济增长预期8.5%左右，充分考虑了我市发展需要和可能，并与全面建成小康社会目标相衔接，符合发展规律，符合信阳实际。我们必须毫不动摇地坚持以经济建设为中心，努力在加快发展、扩大规模的同时，引导各方把主要精力、工作重心放到提质增效和可持续发展上来，放到增加就业和民生改善上来，以实现更高水平、更好质量、更可持续的发展。

　　各位代表！我市与全省、全国一样，经济发展进入了增速换挡、结构调整、动力转换、挑战增多的新常态，但总体向好的基本态势没有改变，蓄势崛起的支撑和后劲日益增强，仍处于大有可为的重要战略机遇期。特别是省委九届八次全会审议通过了《河南省全面建成小康社会加快现代化建设战略纲要》，提出了全面建成小康社会的战略目标、战略方针、战略布局和战略重点，为信阳未来发展指明了前进方向。今年省政府工作报告提出：争取国家编制淮河生态经济带规划、推动高铁沿线城市经济带建设、支持省际交界地区中心城市做大做强、突出抓好“三山一滩”地区扶贫等，为信阳未来发展开辟了广阔空间。郭庚茂书记去年到信阳调研，要求我们注重规律，抓住机遇，后来居上，打好翻身仗。谢伏瞻省长在“两会”期间参加信阳代表团审议，要求我们在加快发展上求突破、在改革开放上闯新路、在塑造生态上画新图、在民生改善上下功夫。省委、省政府的关心支持，为信阳未来发展提供了强大动力。我们必须按照“四个全面”的战略布局，牢牢把握发展主动权，统筹加快经济建设、政治建设、文化建设、社会建设和生态文明建设，协同推进新型工业化、城镇化、信息化、农业现代化和绿色化，确保新常态下有新作为。

　　今年，着重做好九个方面的工作：

　　（一）突出产业集群集聚，加快提升工业化发展水平。更加关注工业、学习工业、研究工业、支持工业，坚定不移地实施产业集聚区“四大工程”、“三大计划”，持续推动产业集聚区上规模上水平上层次，提高吸引力竞争力带动力。提升产业集聚层次。大力支持信阳高新区和潢川经济开发区争创国家级高新区和国家级经济技术开发区，积极推进产业集聚区创新发展。继续坚持集团化体系化引进，集中要素保障，加快推进电子信息产业集聚示范区、信阳国际家居产业小镇、信阳义乌国际商贸城三大项目建设。进一步完善产业集聚区重点项目攻坚督导协调例会制度，加强重点项目联合督查，更加有效地解决发展中的困难和问题，不断提升产业集聚区的产出率、就业率和贡献率，力争全年产业集聚区完成固定资产投资1100亿元，规模以上工业主营业务收入突破1700亿元。扩大主导产业规模。更加聚神聚力聚焦主导产业，壮龙头、建基地、育集群。继续实施重点项目攻坚计划，当年新增重点攻坚项目30个。进一步强化重大项目支撑和配套项目跟进，重点实施150个投资亿元以上的重大结构升级项目，完成投资200亿元以上。大力支持全市规模效益50强企业和50家高成长企业发展，扶持主导产业链上的小微企业成长，加快壮大电子信息、现代家居、绿色食品、现代物流、新型建材、纺织服装等主导产业规模，力争年底主营业务收入超过100亿元的产业集聚区达到12个。提升配套服务水平。继续坚持建设服务与生产服务、项目配套与生活配套、发展保障与社会保障并重，统筹推进产业集聚区基础设施、生产性服务设施和生活性配套设施建设，全面提升产业集聚区承载能力。进一步强化要素保障，做大现有投融资平台，探索支持产业集聚区担保、反担保措施。科学安排计划指标、统筹运用增减挂钩指标，积极推进人地挂钩试点工作，保障产业集聚区项目用地需求。加快创新管理体制，进一步下放管理权限，完善产业集聚区与行政区域套合机制。

　　（二）突出城乡统筹发展，加快推进城镇化发展步伐。以完善设施配套、提升城市品位、增强辐射能力为重点，坚持“一基本两牵动三保障”，推动产业集聚、人口集中、土地集约，加快建设鄂豫皖省际区域性中心城市。优化城镇化空间格局。全面落实我市科学推进新型城镇化实施方案，尽快完成《信阳市城市总体规划（2014－2030年）》的评审报批工作，统筹推进中心城市、县城和中心镇协调发展，加快推进新区开发、老城改造、产业集聚区建设和“6+3”中心城市组团发展，积极争创城乡一体化示范区。加强城市控规专项规划和设计，更好地发挥规划对城市建设的调控、引领和约束作用，积极推进市县“多规合一”。继续围绕建设一个产业集聚区、培育一个百亿元的特色主导产业、打造一个20万以上城市人口的目标，统筹推进产城融合和“两个转移”一齐抓，促进县城做大做强。支持明港镇发展成为具有一定规模的城市。有序推进农业转移人口市民化。坚持以人为核心、以家庭为单元，全面落实户籍制度改革政策和促进农民进城的各项措施，统筹解决农民举家进城的就业、就医、就学、住房等问题，优先解决一批已在城市就业的农村转移人口、棚户区和城中村居民落户城镇，真正让农民进城有希望、生活有奔头、奋斗有回报。提高城镇综合承载能力。继续实施城市基础设施扩能增效工程，加快新老城区的道路衔接，再打通一批断头路，改造提升一批主次干道和背街小巷，着力缓解城市交通拥挤压力。优先发展城市公共交通，再上一批清洁能源公交车辆，进一步实施公共自行车增点扩面，引导市民绿色低碳出行。继续实施一批城市内河治理、污水处理、垃圾处理、供排水、防洪、燃气等基础设施建设，进一步完善城市管网配套。加强城市生态建设，严格执行城市蓝线管理办法，建设海绵城市和生态低碳城市。开展全国文明城市创建工作，持续巩固提升城市创建成果，完善长效机制，全面提高市民综合素质和城市文明程度。加快推进城镇公共服务设施向农村延伸，促进公共服务均等化。深入开展“三违”集中整治，促进房地产业健康发展。建立多元化筹资机制。加大国有资源、资产、资金、资本等整合运作力度，支持各投融资平台通过财政注资、市场募资、整合存量资产、做优增量资产等多种方式提高融资能力；深化与金融机构的战略合作，推进重大城镇项目的批量化、系统化、整体性开发建设；积极探索设立新型城镇化发展基金，支持新型城镇化建设。

　　（三）突出稳粮提效转型，加快转变现代农业发展方式。坚持“三农”重中之重地位不动摇，全面促进强农惠农富农政策落地生根，逐步建立让农业更强、农民更富、农村更美的长效机制。全面深化农村改革试验。抓住信阳被批准为国家级农村改革试验区的机遇，重点深化集体林权制度改革、改进农业补贴办法试点试验、涉农建设性资金整合试验和扶贫开发综合改革试点，重点突破，统筹推进，为全国农村改革试验探索新路子、积累新经验。按照省委、省政府部署，全面完成农村土地承包经营权确权登记颁证工作。统筹抓好“三农”四大工程。持续抓好高标准粮田“百千万”工程，强化水利基础保障作用和科技支撑作用，加强农业综合开发和中低产田改造，再建成高标准粮田75万亩，确保粮食总产稳定在现有水平以上。持续抓好农业产业化集群培育工程，打造一批农业标准化示范区和高标准农产品生产加工基地，培育一批主食产业化和农业产业化重点龙头企业，壮大一批农业产业化集群。持续抓好都市生态农业发展工程，规划建设一批具有综合功能的特色生态养殖园区、标准化种植园区、高新农业科技展示园区和休闲观光农业园区。坚决打好扶贫攻坚战，持续抓好大别山贫困地区群众脱贫工程，实施片区开发和精准扶贫、精准脱贫，全市再实现10.2万农村贫困人口稳定脱贫。加快构建新型农业经营体系。大力发展专业种养大户、家庭农场、农民专业合作社和农业产业化重点龙头企业等新型经营主体，鼓励有条件的农户流转承包土地经营权，发展多种形式的规模经营。充分发挥公益性服务机构基础性作用，积极培育经营性服务组织，加快建立多层次、多形式、多元化的农业社会化服务体系。有序推进新农村建设。坚持“五规合一”，统筹推进“物的新农村”和“人的新农村” 建设齐头并进。加强农村基础设施建设，再解决57.83万农村居民和18.47万农村学校师生的饮水安全问题，新建县乡道路350公里、村道650公里，新增农村沼气用户2450户，改造农村危房1万户，进一步改善农村生产生活条件。持续开展美丽乡村建设，加大古村落保护力度，全面推进农村人居环境综合整治。

　　（四）突出发展现代服务业，加快提升第三产业发展层次。全面落实我市关于加快服务业发展的指导意见，重点实施120个现代服务业重大项目，全面提升服务业发展水平。强化平台支撑。依托商务中心区和特色商业区，加快城市综合体、楼宇经济、专业市场、特色商业街等载体建设，建立健全运营管理和协调推进工作机制，加快“两区”向集聚、特色、服务、融合方向发展。加大招商引资力度，加快培育一批产业高集聚、产出高效益、功能高复合、空间高密度、就业高容量的服务业集群，统筹规划建设一批物流枢纽、信息服务、公共服务、电子商务、科技研发、文化创意、企业孵化等综合服务平台，进一步增强要素吸附能力、产业支撑能力和辐射带动能力，促进现代服务业更好更快发展。放宽市场准入。全面推进工商注册制度便利化，加快推进 “先照后证”改革，降低服务业准入门槛，积极引导和鼓励社会资本平等进入医疗卫生、教育培训、金融保险、健康养老及市政公用事业等领域。抓住国家扩大服务业开放的机遇，在需求旺盛、基础条件好、发展潜力大的领域，再签约一批战略合作项目，引进一批服务业高端人才和先进技术，吸引一批金融机构进驻，落地一批大型企业地区总部和区域性研发、物流、销售、结算、后台服务中心，推动服务业招商由量的增长向质的提升转变。主攻重点产业。大力实施服务业发展“五提升”、“五壮大”工程，促进文化、旅游、现代物流、信息服务、金融保险五大高成长服务业扩容提质，壮大健康养老、教育培训、电子商务、科技服务、物流快递五大新兴服务业规模。尤其在现代物流业方面，以构建区域性现代物流枢纽为目标，加快编制全市现代物流产业发展规划，兴建信阳陆路口岸物流港，建立物流配送中心、冷链运输、冷链储藏等配套体系，培育大型物流企业，力争在一些关键领域形成以信阳为中心或重要节点的现代物流网络；在电子商务方面，加快构建电子交易、电子物流、电子结算三大平台，加强与国内外知名电商的战略合作，培育一批电子商务公共服务平台和线下体验店，支持跨境电商发展。积极构建农村电子商务体系，努力提高工业品下乡、农产品进城的电商贡献率。加强电商培训，着力培养和引进一批电商人才；在文化旅游业方面，加快推进大别山红色生态旅游一体化发展，大力实施鸡公山、灵山联合创建国家5A级旅游景区、南湾湖创建国家生态旅游示范区和新县创建全国旅游标准化试点县工作，持续提升“农家乐”乡村旅游发展，加快建设区域性文化旅游中心。同时，鼓励探索新型商业模式，综合创新供应链、运营销售、服务方式和盈利模式，推动传统服务业转型升级。支持建设一批城市蔬菜便民超市和农村超市。

　　（五）突出全面深化改革，加快释放经济社会发展活力。坚持以经济体制改革为重点，加快构建有利于科学发展的体制机制，努力在重要领域和关键环节取得实质进展和最大综合效益。深化行政体制改革。继续取消和下放一批行政审批事项，基本取消非行政许可审批事项，大幅减少前置审批，编制市级政府部门权责清单。积极探索行政审批标准化，推广网上并联审批模式。今年完成市县政府机构改革和交通运输行政执法体制改革。激发市场主体活力。继续深化国有企业改革，加快国有企业股份制改造，完善企业法人治理结构和内部管理制度；加强国有资产监管，加快市直机关所属企业脱钩改制工作，继续推进国有粮食企业产权制度改革和战略重组，尽快完成信阳国控公司组建工作；落实促进非公经济发展的政策措施，研究制定非公企业进入特许经营领域的具体办法，鼓励非公企业参与国有企业改革，大力支持非公经济、混合型经济发展，切实为各类市场主体创造公平、透明、可预期的市场环境和同等受到法律保护的体制环境。推进财税金融改革。深化预算管理制度改革，继续推进非税收入收缴改革、国库集中收付改革和公务卡改革。推行政府购买社会化服务，扩大政府采购范围和规模。加快推进“营改增”等项改革。建立以政府债券为主体的地方政府举债融资机制，防范和化解债务风险。积极推行政府与社会资本合作模式，完善特许经营制度。大力发展普惠金融，积极推进金融创新，充分利用多层次资本市场、多种金融产品。建立资本市场后备企业库，支持更多企业上市和“新三板”挂牌融资，推动中小企业私募债、区域集优债等企业发债工作。进一步加大招行引资工作力度，积极探索建立金融集聚区，争取吸引更多银行业金融机构入驻信阳。开展农村金融改革试验，探索新农村合作金融发展的有效途径。全面推进县级农信社改制组建农商行工作。鼓励商业银行下沉机构网点。争取实现村镇银行重点乡镇全覆盖。加快推进其他领域改革。深化国有建设用地有偿使用改革，着力解决批而未征、征而未供、供而未用问题。积极稳妥推进水电气等资源性产品价格改革，完善排污收费制度，落实促进节能环保的电价、供热价格、污水处理费等政策，保障基本民生价格稳定。全面推进县级公立医院综合改革，深入推进社会办医，完善全民医保体系，健全重特大疾病保障制度。继续深化科技、教育、文化、体育、供销等体制改革。加快不动产登记制度改革。今年完成党政机关公务用车制度改革。

　　（六）突出扩大对外开放，加快构建开放型经济体系。主动融入“一带一路”合作建设，更加有效地推进新一轮开放合作，构筑对外开放新格局。积极拓宽开放领域。抓住国家放宽外商投资市场准入机遇，加快服务业、农业、基础设施和社会事业等领域开放，建立重点客商跟踪制度，力争引进一批具有战略支撑的重大项目，推动全方位宽领域多层次开放，加快构建开放型经济体系。不断创新招商方式。聚焦主导产业，更加有针对性地制定 “四一”招商行动计划，实施集团化体系化引进战略，开展精准招商，引进缺失链条、补强薄弱链条、提升关键链条，努力形成集群引进和企业抱团转移的倍增效应。着力提升招商质量。切实办好信阳第23届国际茶文化节和第十届豫商大会，力争在开放招商上取得更大成效。进一步强化跟踪服务和项目落地，持续巩固和拓展“中国光彩事业信阳行”等招商成果，提高合同履约率、项目开工率和资金到位率。全面优化开放环境。持续提升服务效能，建立健全客商投资项目审批代理制，进一步提高投资贸易便利化水平；加快构建诚信体系，基本建成市级公共信用信息平台，建立失信行为联合惩戒机制。

　　（七）突出支撑能力建设，加快培育竞争发展新优势。坚持长短结合，科学编制“十三五”规划，深入谋划和实施一批重大项目，争取一批全局性事项列入省和国家规划，做好国家批复大别山革命老区振兴发展规划和编制淮河生态经济带规划前期对接工作。加强基础设施支撑。以构建区域性综合交通枢纽为目标，加快推进宁西客专、京九客专、濮阳至开封至潢川铁路、淮滨至豫皖界高速公路、淮滨至息县淮河航运基础设施等项目前期工作，加快谋划明港至鸡公山高速公路、濮阳至湖北阳新高速公路、鄂豫皖三省大别山北麓高速公路等重大项目，加快实施8条国省干线公路改建工程，开工建设明港机场。以构建“大通关”机制为目标，加快建设信阳保税物流中心等海关特殊监管场所。以构建水利枢纽为目标，积极推进张湾水库、淮河干流一般堤防加固、淮河干流息县水利枢纽等重大水利项目前期工作，全力支持出山店水库建设，继续抓好小型病险水库和大中型病险水闸除险加固、中小河流治理、大中型灌区续建配套与淮干滩区居民迁建和水库移民后期扶持等水利工程建设。以打造坚强电网为目标，大力实施新一轮农村电网改造升级工程，解决长期困扰农村地区的低电压问题。积极协调配合做好宁东—绍兴特高压直流输电工程建设。加强科技创新支撑。强化企业创新主体地位，支持企业加强与央企、科研院所、高等院校深度合作，促进产业技术创新战略联盟协同创新，引进或共建一批创新平台，实施一批省级重大科技专项，突破一批制约我市主导产业链式发展的关键核心技术。全面推进质量立市战略，支持羚锐、华英等龙头企业创建国家级技术研究中心、工程（技术）研究中心和工程重点实验室，积极申报建设国家茶叶质检中心、国家岩石黏土类非金属矿产品质检中心及省级家具质检中心，推动产业不断创新升级。继续创新人才培养选拔、评价使用、流动配置、激励保障机制，着力创造有利于吸引人才、培养人才、留住人才的环境，引导科技人才、工程技术人才向产业一线集聚。厚植创新文化，完善创新机制，让大众创业、万众创新的活力竞相迸发。加强信息化支撑。加快智慧城市、无线城市、光网城市等工程建设，加快 “宽带信阳”、“三网融合”建设步伐，统筹推进城乡宽带网络升级改造，扩大4G网络技术规模化商用，推动大数据、物联网、云计算等在民生和社会领域应用。积极建设覆盖城乡人口、建设、治安、环保等领域的管理应用平台，以信息化促进城乡建管协调发展。大力发展电子政务，全面实施信息惠民工程，统筹教育、医疗卫生、交通和社会保障等信息化建设，提高社会治理领域信息互通和资源共享水平。

　　（八）突出改善环境质量，加快推进生态文明建设。环保执法必须硬起手腕，以更大的决心和力度推进绿色循环低碳发展，共建青山常在、清水长流、空气常新的美丽家园，努力让信阳更具吸引力、竞争力。加强环境保护。深入开展国家主体功能区试点示范市建设，大力实施大别山生态安全屏障和淮河上游水生态建设工程，加强淮河源生物多样性保护与可持续利用，持续强化南湾湖国家重点湖泊生态保护和环境治理，加快启动出山店水库区域的统一规划和生态环境保护工作。深入开展全国绿化模范城市创建工作，大力实施林业生态市建设提升工程，持续提高城区绿化覆盖率和山区森林覆盖率，形成大面积、高容量的绿色开敞空间，构筑以森林植被为主体、布局合理、功能完善的森林生态体系和生态安全屏障，提高生态承载能力。全年完成造林26.2万亩、森林抚育43.9万亩。推进综合治理。继续实施“蓝天工程”、“碧水工程”和“乡村清洁工程”，加强饮用水水源地保护，加大工业污染源治理，推进机动车尾气治理、城市施工和道路扬尘防治、秸秆焚烧整治，深化畜禽养殖密集区环境综合治理，加强重点行业、重点部位、重点区域污染防治，不断改善和提高环境质量。落实最严格的水资源管理制度，建立覆盖市县的“三条红线”控制指标体系，建设节水型社会。强化节能减排。落实环境保护负面清单，实行更加严格的环境准入制度，完善能源消费总量和重点污染物排放总量预算管理制度，实施排污权、碳排放、水权、林权交易制度，用好生态环境补偿机制，增强企业、公共机构和全社会节能减排内生动力。进一步完善政府绩效考核办法，实行最严格的源头保护、损害赔偿和责任追究制度，坚决向污染宣战到底，坚决让损害环境者付出代价。

　　（九）突出强化民生保障，加快构建和谐社会。坚持民之所需，政府所为，继续抓好省“十项重点民生工程”和市“十件实事”，集中财力办好群众急需实用的事情。更加重视就业创业。坚持把就业作为经济社会发展的优先目标，实施更加积极的就业政策，继续抓好高校毕业生、农村转移劳动力、城镇困难人员、退役军人等就业工作，妥善解决化解产能过剩中下岗分流职工再就业问题，动态消除“零就业家庭”。深入推进全民技能振兴工程，加强职业技能培训，推动实现更高质量就业。充分发挥创业带动就业的倍增效应，支持建设一批创业孵化示范园区，以创业促进就业、引领就业。完善“三市一库”就业服务保障体系，统筹解决招工难与就业难问题。加大治理欠薪和打击非法中介力度，加强劳动保障监察和劳动人事争议调解仲裁工作，构建和谐稳定的劳动人事关系。更加重视社会保障。坚持保基本、兜底线、促公平、可持续，进一步完善各项社会保险和社会救助制度，扩大社会保障覆盖范围，提高社会保险统筹层次，确保各类社会保障资金按时足额发放。稳步推进机关事业单位养老保险制度改革，全面实施城镇居民大病保险制度，继续提高城乡居民养老保险和企业退休人员基础养老金，提高新农合、城镇居民医保财政补助标准和基本公共卫生服务经费，提高城乡低保、农村五保和优抚对象补助标准。加快推进社会保障一卡通。加强社会养老服务体系和社会福利中心建设。大力发展慈善、残疾人、妇女儿童、红十字、老龄等事业。健全农村留守儿童、妇女、老年人关爱服务体系。扎实推进保障性安居工程建设，争取全年新开工保障性住房5978套，基本建成12218套。更加重视社会事业。加强基层医疗人才培养和医疗卫生机构标准化建设，努力规划建设一批新的医院，完善医疗卫生服务体系。着力解决过度医疗问题，妥善处置医疗纠纷，构建和谐医患关系。坚持计划生育基本国策，推进免费孕前优生健康检查项目城乡全覆盖。构建普惠性基础教育体系，继续推进第二期学前教育三年行动计划，深入实施全面改造薄弱学校和扩充城镇义务教育资源五年计划，不断扩大普通高中优质教育资源，推进义务教育均衡发展和普通高中多样化发展。面向市场需求，大力发展现代职业教育，落实普惠性中等职业教育政策，从秋季起对全日制中职在校生全免学费。加强教师队伍建设。办好人民满意的教育。深入推进精神文明建设，持续弘扬焦裕禄精神和大别山精神，培育和践行社会主义核心价值观。大力繁荣文化事业，深入开展精神文明建设“五个一工程”和何景明文学奖评选活动，倡导全民阅读，大力发展档案、史志、文史事业，编撰出版《信阳历史文化丛书》，加强文化遗产保护与合理利用，努力打造一批文化精品。深入实施广播电视户户通等文化惠民工程，加强公共文化体育设施建设，推动全民健身运动开展，办好第七届全国公路自行车公开赛。更加重视社会建设。牢固树立“大平安”理念，着眼“双安”、推进“双治”、强化“双基”，完善四项基层基础制度，加快从传统社会管理向现代社会治理转变。深入开展“信访法治建设年”活动，完善“四有”工作机制和民声手机短信平台，抓好依法逐级走访、诉访分离和积案化解工作。建立健全食品药品安全监管体系，加强从田间到餐桌的全程监管，确保食品安全。严格落实安全生产责任，加强公共安全体系和企业安全生产基础建设，深化重点行业和领域整治，坚决遏制重特大事故发生。创新立体化社会治安防控体系，构建“一村一警”长效机制，严厉打击刑事犯罪和黑恶势力。加强法律援助和司法救助工作。依法打击非法集资。建立健全应急管理体系、重大决策社会稳定风险评估机制和各类风险化解机制，确保社会和谐稳定。

　　继续加强新形势下国防教育和国防后备力量建设，支持驻信解放军、武警部队、预备役部队、民兵和人民防空建设，深入开展双拥模范城创建活动，巩固军政军民团结，促进军民融合深度发展。大力支持人民团体发挥桥梁纽带作用，认真做好民族、宗教工作，切实加强外事、侨务、对台工作。继续抓好机关事务、地质、气象、地震等各项工作。

　　各位代表！今年，在完成省定“十项重点民生工程”的同时，继续抓好“十件实事”，具体安排意见已经印发，请予审议。

　　三、全面加强政府自身建设

　　做好政府工作必须加强自身建设，提升政府治理水平。

　　（一）全面加强服务型政府建设。进一步强化政府公共服务、市场监管、社会管理、环境保护等职责，推动政府职能加快向创造良好发展环境、提供优质公共服务、维护社会公平正义转变。进一步提高政府公共服务效能，推动公共资源更多地向民生领域倾斜，努力为人民群众提供更好、更优、更均衡的公共产品服务，切实解决好关系群众切身利益问题，真正打通联系群众“最先一公里”和服务群众“最后一公里”，切实让老百姓少跑腿、少操心、少费事。进一步加强政务诚信建设，以政务诚信带动商务诚信和社会诚信，提高政府公信力和执行力。

　　（二）全面加强法治型政府建设。深入开展法制宣传教育,大力弘扬法治精神，形成对法律敬畏和法律信仰的良好氛围。严格推行权力清单制度，坚持法无授权不可为、法定职责必须为，全面推进政府工作法治化。健全依法科学民主决策机制，加强决策合法性审查，落实重大决策终身责任追究制度。深化服务型行政执法，落实行政执法责任制，加强执法监督，严格规范公正文明执法。自觉接受人大及其常委会法律和工作监督，主动接受政协民主监督，严格执行人大及其常委会的决议决定，认真办理人大代表建议和政协提案，广泛听取民主党派、工商联、无党派人士和人民团体意见，接受新闻舆论和社会公众监督，扩大政务公开范围，让权力在阳光下运行，让人民更好地监督政府。

　　（三）全面加强创新型政府建设。主动适应经济新常态，加 快创新政府管理理念和方式，优化政府机构设置、职能配置和工作流程，健全决策、执行、监督和问责机制，切实做到宽政策、强服务、优环境。积极创新服务方式，提供基本公共服务尽量采用购买服务方式，第三方可提供的事务性管理服务交给市场或社会去办。创新公务员管理机制，加强公务员教育培训，着力打造一支敢于创新、敢于担当、敢于负责的公务员队伍，坚决纠正不作为、乱作为，坚决克服懒政、怠政，坚决惩处失职、渎职，确保政令畅通、政策落地。

　　（四）全面加强廉洁型政府建设。严明政治纪律，严守政治规矩，严格落实党风廉政建设主体责任和“一岗双责”，切实把廉洁从政贯穿到政府工作全过程。严格落实中央八项规定，扎实推进“三严三实”专题教育，坚持不懈纠正“四风”，推进作风建设常态长治长效。全面加强行政监察和审计监督，进一步完善财政资金支出、工程项目招投标、国有资源交易等各项制度，切实扎牢制度笼子。始终保持反腐高压态势，坚决对腐败分子零容忍、严查处。坚持勤俭办一切事情，严控“三公”经费，切实把有限财力投入到保障发展、改善民生上，使发展成果更多更公平地惠及人民群众。

　　各位代表！新的一年面临新的机遇，新的一年充满新的希望。让我们紧密团结在以习近平同志为总书记的党中央周围，在市委的坚强领导下，肩负起全市人民的重托，凝聚起全市人民的智慧力量，持续求进，务实发展，积极作为，努力完成全年经济社会发展目标，为我市全面建成小康社会、加快现代化建设奠定坚实基础！
